# Supplementary material for: Temporal Trends in Apparent Food Consumption in Bangladesh: A Joinpoint Regression Analysis of FAO’s Food Balance Sheet Data from 1961 to 2013
Source: Nutrients. 2019 Aug 10;11(8):1864. doi: 10.3390/nu11081864 (PMC6723082; doi:10.3390/nu11081864)
Supplement: Supplementary file 1 [file nutrients-11-01864-s001.pdf]

Supplementary Table S1

Table S1: Food items grouped into the eleven food groups

Food groups	Food items	Comment
**Cereals**	Rice<br>Wheat and products<br>Barley and products<br>Maize and products<br>Millet and products<br>Sorghum and products<br>Cereals, other<br>Oats	
**Starchy roots**	Potatoes and products<br>Sweet potatoes<br>Cassava and products<br>Roots, others	
**Pulses**	Pulses, other and products<br>Beans<br>Peas	
**Fish**	Freshwater fish<br>Demersal fish[1]<br>Pelagic fish[2]<br>Marine fish, other<br>Crustaceans[3]	Cephalopods[4] and the Molluscs, other these two goups have zero food supply (g/day/person) from 1961 to 2013.
**Eggs**	Eggs	No categorization is given[5]
**Meat**[6]	Bovine meat<br>Mutton and goat meat<br>Poultry meat<br>Meat, other	Pig meat has zero food supply (g/day/person) from 1961 to 2013.
**Vegetables**	Vegetables, other<br>Tomatoes and products<br>Onions	
**Fruits**	Orange, mandarins<br>Lemon, limes and products<br>Grapefruit and products<br>Citrus, other<br>Bananas<br>Apples and products<br>Pineapples and products<br>Grapes and products<br>Dates<br>Fruits, other	

**Milk**	Milk excluding butter	No categorization is given[5]
**Vegetable oils**	Soya bean oil<br>Ground nut oil<br>Sunflower seed oil<br>Rape and mustard oil<br>Cottonseed oil<br>Palm oil<br>Coconut oil<br>Sesame seed oil<br>Rice bran oil<br>Oil crops oil, other	Olive and Maize germ oil have zero food supply (g/day/person) from 1961 to 2013
**Sugar**	Sugar non-centrifugal[7]<br>Sugar (raw equivalent)<br>Sweeteners, other	Honey has zero food supply (g/day/person) from 1961 to 2013

[1]Demersal fish are fishes that live and feed near the bottom of seas and lakes such as eel and North American catfish etc. [2]Pelagic fish live in the pelagic zone of the ocean or lakes, neither close to the bottom nor near the shore such as tuna, pomfret, and mackerels etc. [3]Crustaceans includes crabs, shrimps, lobsters, and prawns. [4]Cephalopods included squid and octopus. [5]Eggs and milk groups have no specific further categorization in the FAOSTAT database. [6]Meat and offal consumption were added to make this one group. [7] Non-centrifugal sugars include jaggery and gur.
